# Predictive Value of S100-B and Copeptin for Outcomes following Seizure: The BISTRO International Cohort Study

**DOI:** 10.1371/journal.pone.0122405

**Published:** 2015-04-07

**Authors:** Yonathan Freund, Benjamin Bloom, Jerome Bokobza, Nacera Baarir, Said Laribi, Tim Harris, Vincent Navarro, Maguy Bernard, Rupert Pearse, Bruno Riou, Pierre Hausfater, the BISTRO investigators

**Affiliations:** 1 Sorbonne universités, UPMC Univ Paris 06, UMRS INSERM 1166, IHU ICAN, Paris, France; 2 Emergency Department, Hôpital Pitié-Salpêtrière, Assistance Publique-Hôpitaux de Paris (APHP), Paris, France; 3 Emergency Department, Royal London Hospital, London, United Kingdom; 4 Emergency Department, Hôpital Tenon, APHP, Paris, France; 5 Emergency Department, Hôpital Lariboisière, APHP, Paris, France; 6 Neurology Department, Hôpital Pitié-Salpêtrière, APHP, Paris, France; 7 Biochemistry Department, Hôpital Pitié-Salpêtrière, APHP, Paris, France; 8 Adult critical care unit, Royal London Hospital, London, United Kingdom; 9 Université Paris 5 Paris Cité, Paris, France; 10 Queen Mary University of London, London, United Kingdom; Sainte-Anne Hospital Center, FRANCE

## Abstract

**Objective:**

To evaluate the performance of S100-B protein and copeptin, in addition to clinical variables, in predicting outcomes of patients attending the emergency department (ED) following a seizure.

**Methods:**

We prospectively included adult patients presented with an acute seizure, in four EDs in France and the United Kingdom. Participants were followed up for 28 days. The primary endpoint was a composite of seizure recurrence, all-cause mortality, hospitalization or rehospitalisation, or return visit in the ED within seven days.

**Results:**

Among the 389 participants included in the analysis, 156 (40%) experienced the primary endpoint within seven days and 195 (54%) at 28 days. Mean levels of both S100-B (0.11 μg/l [95% CI 0.07–0.20] vs 0.09 μg/l [0.07–0.14]) and copeptin (23 pmol/l [9–104] vs 17 pmol/l [8–43]) were higher in participants meeting the primary endpoint. However, both biomarkers were poorly predictive of the primary outcome with a respective area under the receiving operator characteristic curve of 0.57 [0.51–0.64] and 0.59 [0.54–0.64]. Multivariable logistic regression analysis identified higher age (odds ratio [OR] 1.3 per decade [1.1–1.5]), provoked seizure (OR 4.93 [2.5–9.8]), complex partial seizure (OR 4.09 [1.8–9.1]) and first seizure (OR 1.83 [1.1–3.0]) as independent predictors of the primary outcome. A second regression analysis including the biomarkers showed no additional predictive benefit (S100-B OR 3.89 [0.80–18.9] copeptin OR 1 [1.00–1.00]).

**Conclusion:**

The plasma biomarkers S100-B and copeptin did not improve prediction of poor outcome following seizure. Higher age, a first seizure, a provoked seizure and a partial complex seizure are independently associated with adverse outcomes.

## Introduction

Patients attending the emergency department (ED) with seizure account for 0.5 to 7% of all ED visits, and approximately one million visits per year in the United States [1–5]. The impact of one or more seizures on an individual includes the potential for physical trauma, time off work, degeneration into status epilepticus and the risk of a life threatening acute anoxic event [6–8]. Therefore the ability to risk assess for recurrence is of critical importance.

The rate of long term recurrence is high, with a three year risk of 30% after acute symptomatic seizures and 50 to 70% after an unprovoked seizure [9–12]. The rate of early seizure recurrence (ESR) is less well established. ESR rates have been reported to be 19% in the first 24 hours, and up to 30% in cases of alcohol related seizure [4,13]. One prospective study has evaluated predictors of ESR, and found that alcoholism, low plasma glucose, and a Glasgow coma scale (GCS) less than 15 were independently associated with a higher risk of ESR [13]. As the risk of other adverse events, such as hospitalisation or death, following a seizure have not been studied, there may be further variables in addition to the three identified that can assist in the risk stratification of patients presenting to the ED with seizure.

The astroglial S100-B protein is a specific marker of cerebral injury. Raised S100-B has value in predicting adverse neurological outcomes in cardiac arrest and traumatic brain injury [14–16]. S100-B concentration is normal following febrile seizure in children. That febrile seizures are considered to be relatively harmless contributes to the hypothesis that elevated S100-B might predict adverse neurological outcomes [17,18]. Copeptin, the c-terminal part of the vasopressin molecule, is a biomarker of endogenous stress. Recently, it has been described as a good prognostic marker in neurological disorders, such as traumatic brain injury [19], intracerebral hemorrhage, and stroke [20,21].

We hypothesised that these two biomarkers may have an incremental added prognostic value to routine clinical data to predict adverse events following seizure related ED visits.

## Methods

### Study design, setting and participants

The Biomarkers In Seizure To predict Recurrences and severe Outcomes (BISTRO) is a prospective international cohort study (NCT01774500), conducted from January 2013 to December 2013. The primary objective is to establish the incremental value of combining S100-B and copeptin levels with standard clinical variables to identify patients most at risk of complications following presentation in the ED with seizure.

We enrolled patients from four centres: one in London, UK and three in Paris, France. Participants’ informed signed consent was sought prior to enrolment, and institutional review boards from both countries approved the study (Comité de protection des personnes—Paris Ile de France 6, Paris, France; and NHS Health Research Authority, National Research Ethics Service Camberwell St Giles, United Kingdom). In cases in which informed consent could not be obtained from the patient due to a decreased level of conscious, a next-of-kin signed informed consent was mandatory prior to enrolment. After the patient returned to a normal level of consciousness, their signed informed consent was then sought. When this was not obtained, the patient was excluded from the study.

The study design and report is in accordance with the STROBE statement [22]. Patients were eligible to become study participants if they were 18 years or older and had had one or more convulsive seizures within 24 hours. Patients were excluded if they were less than 18 years; pregnant; prisoners; and those for whom seven or 28 day follow up was deemed impossible. Patients were screened in real time in the EDs of the participating centres.

### Outcomes

The primary endpoint was a composite endpoint of seizure recurrence, or all cause death, hospitalisation, or rehospitalisation or return visit in the ED within seven days.

Secondary endpoints included seizure recurrence at seven and 28 days; ICU admission; death within seven and 28 days; and length of hospitalization within seven and 28 days. The decision to hospitalise a patient depends on individual physicians and as such may be considered subjective. To reduce the effect of this subjectivity, a sensitivity analysis was run with a modified primary endpoint that excluded those patients that were hospitalised for less than 24 hours. Finally, as predicting adverse events in discharged patients is of great importance, we ran a sub-analysis focusing only on patients that were not admitted after their first ED visit.

### Variables

Clinical and physiological data were recorded; white cell count, sodium, calcium, glucose, and lactate were routinely measured within the participating centres. Venous blood samples were taken in heparinised tubes to measure S100-B and copeptin. The sample for S100-B and copeptin was frozen at -80°C and all samples were measured in a single batch at the end of the study to avoid bias from assay discrepancy. The assay for copeptin measurement was performed on a KRYPTOR analyzer using the commercial sandwich immunoluminometric assay (B.R.A.H.M.S Aktiengesellschaft, Hennigsdorf, Germany). The lower detection limit is 4∙8 pmol/L, and the functional assay sensitivity is < 12 pmol/L. The limit of quantification (10% coefficient of variation [CV]) is 14∙1 pmol/L. In our laboratory, the CV were found to be <5% (4∙4% at 28∙86 pmol/L and 4∙6% at 95∙84 pmol/L). S100-B measurement was performed on an Elecsys (Roche Diagnostics, Mannheim, Germany). The lower detection limit is 0∙005 μg/L and the functional assay sensitivity is 39 μg/L. In our laboratory, the CV was found to be <5%. Copeptin and S100-B determinations were performed blinded to the clinical assessment of the emergency physicians. Follow up was performed either by telephone or hospital visit.

Since the definition of “epilepsy” is controversial, and has varied in recent years [23–25], a patient was considered epileptic if a neurologist had ever diagnosed the condition, if the patient had an unprovoked seizure and evidence of remote CNS lesion or if the patient was currently on antiepileptic drug. A remote lesion is a CNS lesion that is stable and is not acute (for instance a stroke sequellum).This approach is in accordance with recommendations from the International League Against Epilepsy (ILAE) for a pragmatic definition of epilepsy [25]. A seizure was classified, according to ILAE guidelines, as provoked if it could have been related to an acute systemic insult or acute CNS lesion (there are many causes for a provoked seizure, for instance alcohol intoxication, alcohol withdrawal, hypoglycemia) occurring within the previous seven days, or unprovoked if not. Unprovoked seizures were classified as idiopathic, or remote symptomatic in the presence of a known CNS lesion. Seizures in the setting of sleep deprivation were not considered provoked [26].

Patients were followed up for 28 days, and were called (or visited if still in the hospital) at day seven and 28 to assess endpoints. Participants with missing data regarding the two biomarkers, and participants lost to follow up were excluded.

### Study size

On the basis of pre-existing literature, we estimated the rate of the primary endpoint at day seven to be 20%. To avoid overfitting and in order to be able to include at least 10 variables in the logistic regression model, there needed to be at least 100 events in our sample [27]. Furthermore, this minimal number of 100 events is warranted for external validation [28]. Therefore a total sample size of 500 participants was required for this study. An interim analysis of outcome showed a higher rate of the endpoint than expected (35%), which reduced the required sample size to 350 participants.

### Statistical analysis

Data are expressed as mean ± standard deviation (SD) for Gaussian variables; median and 25 to 75% interquartile range for non-Gaussian variables; and number and percentage for categorical variables with 95% confident interval. Normality was assessed using the Kolmogorov-Smirnov test. Measures of diagnostic accuracy were calculated with their 95% confidence intervals (CI) for S100-B and copeptin. Receiving operator characteristics (ROC) curves were constructed and their area under the curve was calculated. Thresholds were determined using the Youden’s method. Comparison of the two groups was performed using the Student t test, the Mann-Whitney U test, and Fisher's exact method when appropriate.

A multiple logistic regression was performed to assess independent variables associated with the primary endpoint, and odds ratios (ORs) with their 95% CI were calculated. To avoid overestimation, a conservative approach was used and all clinically relevant variables were included [29]. These variables were determined a priori upon previous literature and clinical relevance (namely age, first seizure, history of epilepsy, neuromuscular impairment, chronic alcohol intake, focal neurological deficit, complex partial seizure, provoked seizure, GCS < 15, body temperature > 37∙5°C) and the two studied biomarkers, S-100 and copeptin. Correlation between all variables were calculated, and in case of a coefficient of correlation R^2^>0∙6, only the most clinically significant variable was entered in the model. Calibration of the model was estimated with Hosmer-Lemeshow test, and discrimination with the c-index. Internal validation was assessed using the bootstrap resampling method (n = 500, without replacement) [30]. To present the internal validation,the difference (optimism) between the c-statistics observed in the population and in the bootstrapped sample was calculated [30].

All analyses were performed using SPSS software (IBM, Armonk, NY), all comparisons were two-tailed and a p value of 0∙05 was required to reject the null hypothesis. The statistical plan was decided before the onset of the study.

## Results

**Fig 1:**
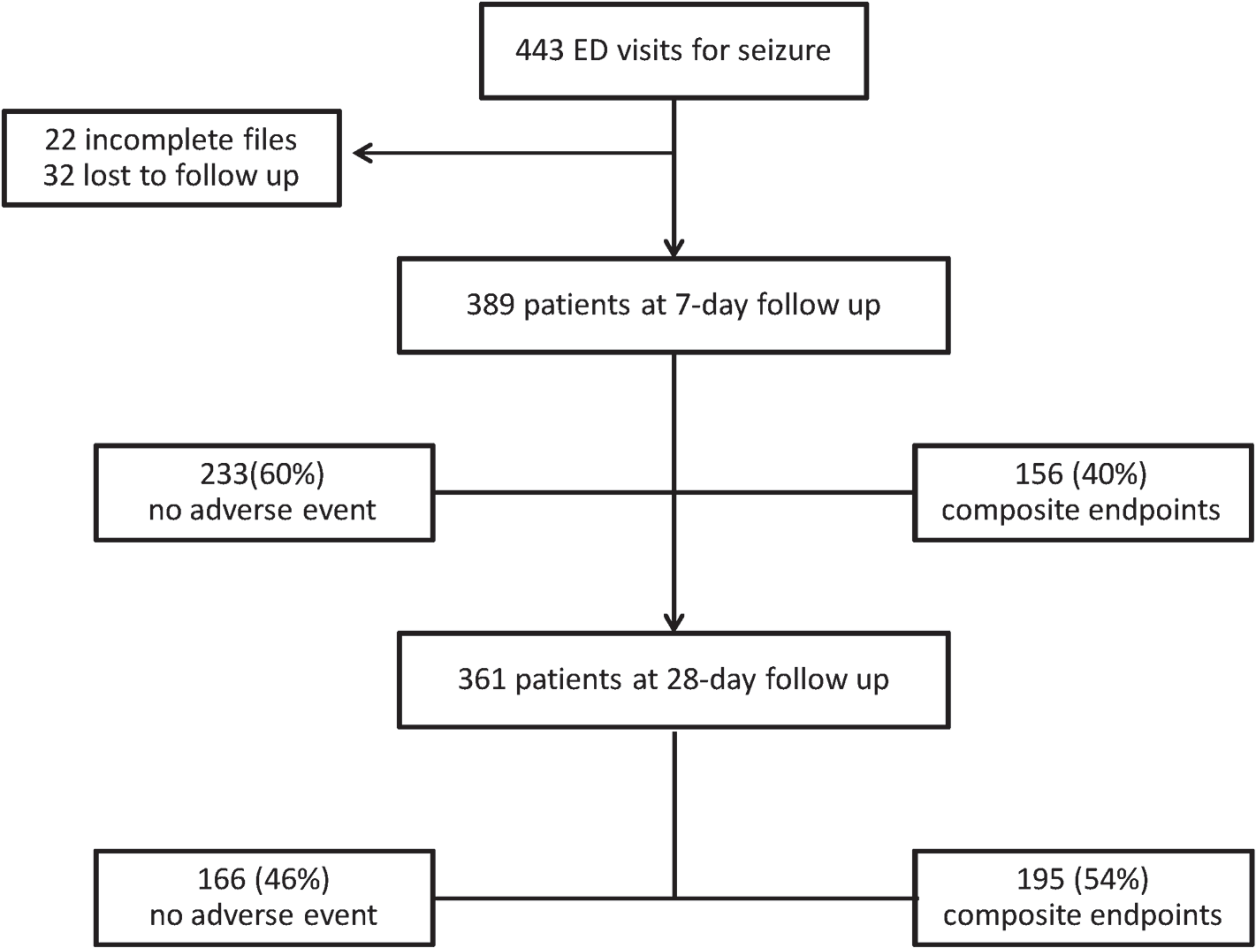
Flow chart ED: emergency department. Composite endpoint of recurrence, hospitalization or death at day seven.

In the period of inclusion, 443 participants were enrolled. Twenty two participants had no S100-B and copeptin measurements, and 32 were lost to follow up (Fig 1). Therefore 389 participants were included in the analysis, of which 87 (22%) were from the United Kingdom and 302 (78%) from France. The mean age of the studied population was 44 years (SD 18), and 58% were male. One hundred and thirty (33%) presented to the ED with a first seizure and 259 (67%) were considered epileptic according to the definition above. Main baseline characteristics are summarized in Table 1.

**Table 1:** Characteristics of study cohort.

		**Total**	**All patients**		**No event in 7 days**		**Recurrence or severe outcome at day 7**	
		389	389		233	(60%)	156	(40%)
**Characteristic**	Age, mean (SD), y		44	(18)	40	(16)	51	(20)
	Sex Male, No. (%)		229	(58%)	143	(61%)	86	(55%)
	Sex Female, No. (%)		160	(42%)	90	(39%)	70	(45%)
	Seizure in the ED, No. (%)		73	(19%)	21	(9%)	52	(33%)
	Seizure		259	(67%)	170	(0.72)	89	(57%)
**Past Medical History, No. (%)**	Epilepsy		217	(56%)	147	(63%)	70	(45%)
	Stroke		32	(8%)	16	(7%)	16	(10%)
	Meningitis		14	(4%)	7	(3%)	7	(4.5%)
	Neuromuscular impairment		22	(6%)	7	(3%)	15	(10%)
	Chronic alcohol intake		50	(13%)	20	(9%)	30	(20%)
	Drug		13	(3%)	8	(3%)	5	(3%)
	Benzodiazepin		56	(14%)	37	(16%)	19	(12%)
**Current medication, No. (%)**	Anti epileptic drug		172	(44%)	114	(49%)	58	(37%)
	Headache		100	(26%)	62	(27%)	38	(24%)
**On Examination, No. (%)**	Photophobia		13	(3%)	9	(4%)	4	(3%)
	Confusion		43	(11%)	14	(6%)	29	(19%)
	Neurological deficit		13	(3%)	1	(0.5%)	12	(8%)
	Partial simple		24	(6%)	13	(6%)	11	(7%)
**Type of seizure, No. (%)**	Complex partial		41	(10%)	13	(6%)	28	(18%)
	Generalised tonic clonic		290	(75%)	179	(77%)	111	(71%)
	Absence		31	(8%)	22	(9%)	9	(6%)
	Acute Symptomatic		67	(17%)	17	(7%)	50	(32%)
	Remote symptomatic		49	(13%)	25	(11%)	24	(15%)
	Idiopathic		273	(70%)	191	(82%)	82	(53%)
	Witnessed		280	(72%)	160	(69%)	120	(77%)
	Time from Seizure to ED visit, median [IQR], hours		1.5	[1–2]	1.5	[1–2]	0.7	[0–2]
	Heart rate, mean (SD)	378	89	(19)	89	(19)	90	(17)
**Physiological parameters on admission**	Systolic BP, mean (SD)	380	129	(21)	129	(19)	129	(24)
	Diastolic BP, mean (SD)	380	77	(15)	77	(13)	79	(17)
	Temperature, mean (SD)	376	36.6	(0.6)	36.6	(0.5)	36.8	(0.7)
	GCS, median [IQR]	379	15	[15–15]	15	[15–15]	15	[15–15]
	GCS<15, No (%)	389	45	(12%)	25	(11%)	20	(13%)
	Pulse oxymetry, median [IQR]	380	97	[96–99]	98%	[96–99]	97%	[95–99]
	WBC (Giga/l), median [IQR]	325	9.8	[7.0–13]	9.5	[6.5–12.7]	10.4	[7.4–13]
**Laboratory results**	Glucose (mmol/l), median [IQR]	270	6.1	[5.2–7.3]	5.8	[5.1–6.8]	6.4	[5.4–8]
	Sodium (mmol/l), mean (SD)	365	137	(12)	137	(13)	137	(11)
	Calcium (mmol/l), median [IQR]	289	2.3	[1.3–2.4]	2.4	[2.3–2.5]	2.3	[1.2–2.4]
	Lactate (mmol/l), median [IQR]	176	1.9	[1.2–3.6]	1.65	[1.2–3.3]	2.1	[1.3–3.7]
	S100B (μg/l), median [IQR]	389	0.10	[0.07–0.16]	0.09	[0.07–0.14]	0.11	[0.07–0.2]
	Copeptin (pmol/l), median [IQR]	389	19	[8–54]	17	[8–43]	23	[9–104]

SD, standard deviation; IQR, 25–75% interquartile range; ED, emergency department; GCS, Glasgow coma scale; WBC, white blood cells. All laboratory results were obtained from venous blood.

One hundred and fifty six participants (40%) experienced the primary endpoint of death, hospitalization, seizure recurrence, rehospitalisation or return visit to the ED within seven days and 195 (54%) at 28 days. The primary endpoint occurred in 56%, 40%, 31% and 26% in participants from Royal London Hospital, Pitié-Salpêtrière, Tenon and Lariboisière hospitals respectively (p = 0∙003 for UK vs France). Sixty patients (15%) had a seizure recurrence within seven days. Main outcomes are summarized in Table 2.

**Table 2:** Outcomes and follow up of the study cohort.

		**Total**	**All patients**	
**Disposition from ED**	Home	**389**	243	(63%)
	Observation unit		95	(29%)
	Hospitalisation		126	(32%)
	Admission in ICU		11	(3%)
	Admission in neurosurgery		15	(4%)
	Death		2	(1%)
**Follow up day 7**	Seven days free of hospital	**389**	224	(58%)
	Recurrence		60	(15%)
	Re hospitalisation		16	(4%)
	Number of hospital free days, median [IQR]		7	[4–7]
	ICU admission		14	(4%)
	Death		5	(1%)
**Follow up day 28**	28 days free of hospital	**361**	185	(51%)
	Recurrence		97	(27%)
	Rehospitalisation		29	(8%)
	Hospital free days, median [IQR]		28	[25–28]
	ICU admission		16	(4%)
	Death		10	(2%)

ED, emergency department, ICU, intensive care unit; IQR, 25–75% interquartile range.

**Fig 2:**
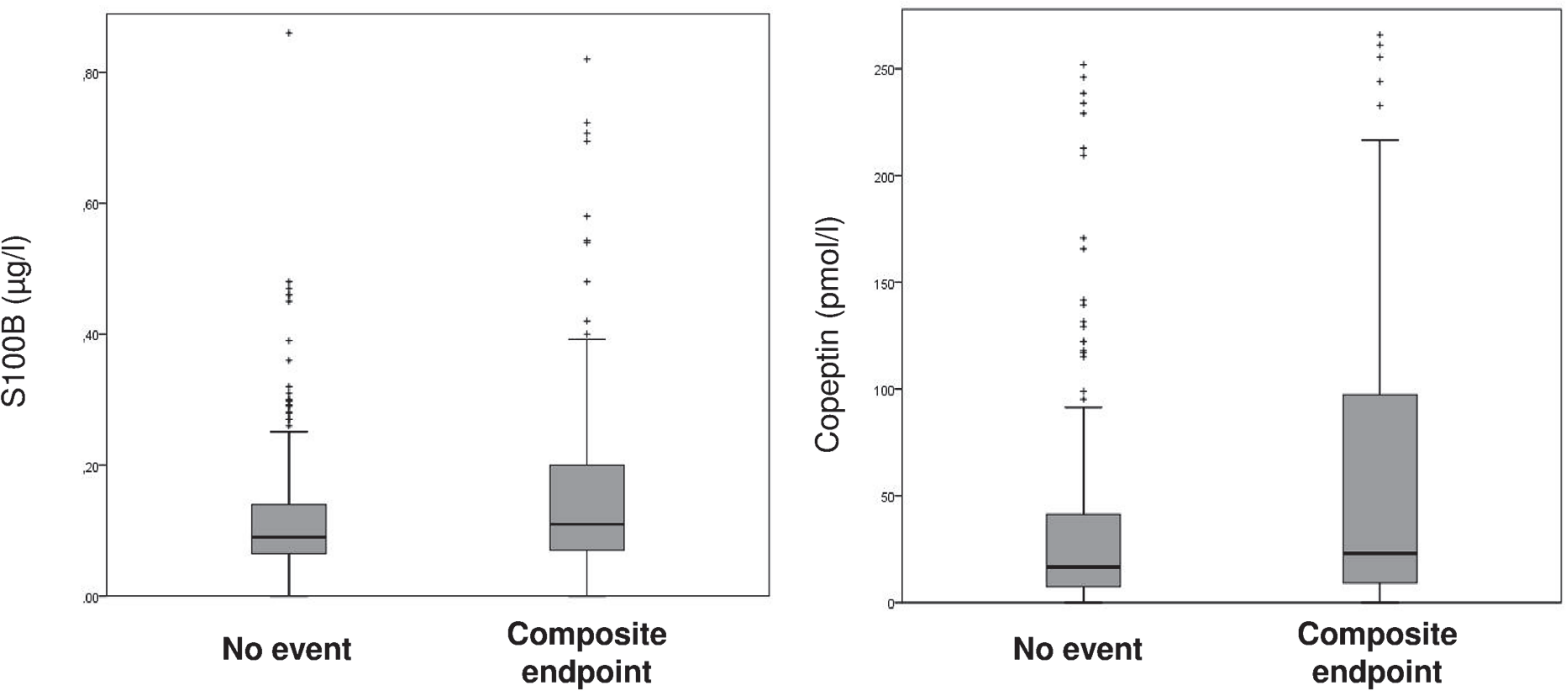
S100B and copeptin values in the two groups. Box plot with median, interquartile range, and 5^th^ and 95^th^ centile. Composite endpoint of recurrence, hospitalization or death at day seven.

Copeptin and S100-B were significantly higher in participants that experienced the primary combined endpoint than in the others: 0∙11 [0∙07–0∙20] vs 0∙09 [0∙07–0∙14] μg/l (p = 0 02) for S100-B and 23 [9–104] vs 17 [8–43] pmol/l (p<0∙001) for copeptin (Fig 2).

**Fig 3:**
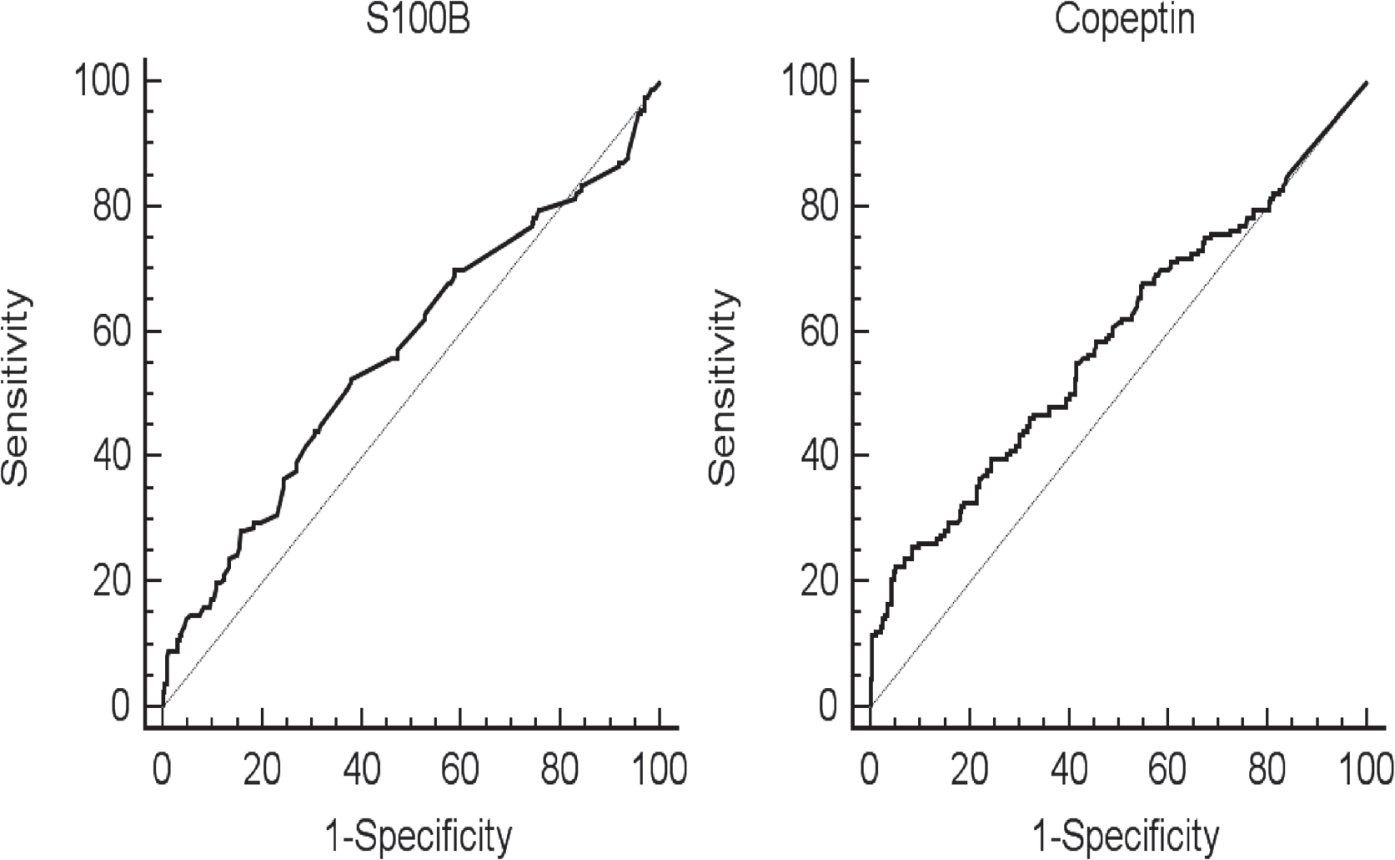
Receiving operator characteristics curve for Copeptin and S100B. Area under the curve 0.57 [95% CI 0.51–0.64] for S100B, p = 0.01, and 0.59 [95% CI 0.54–0.64] for copeptin, p = 0.02.

ROC curves for S100-B and copeptin are reported in Fig 3, with a respective area under the curve of 0∙57 [95% CI 0∙51–0∙64] and 0∙59 [95% CI 0∙54–0∙64] (p<0∙05 for both). Using Youden’s method, a threshold value of 0∙1 μg/l for S100-B and 100 pmol/l for copeptin was found, which corresponded to a sensitivity and specificity of 57% [49–65%] and 53% [46–59%] respectively for S100-B, and 24% [18–31%] and 92% [88–95%] respectively for copeptin. Complete diagnostic performances are reported in Table 3 with different thresholds. Of note, we studied “positive S100 AND positive copeptin”, as well as “Positive S100 OR positive copeptin”, with different thresholds and we found that no combination resulted in satisfactory diagnostic performances (data not shown). When considering more homogenous populations, for example epileptic patients, or patients with provoked seizure, neither of these two biomarkers showed good diagnostic performances (data not shown).

**Table 3:** Diagnostic performances of S100-B and Copeptin, and 95% confidence interval.

Biomarker	Threshold	Sensitivity		Specificity		PPV		NPV		LR+		LR-	
S100-B	0.1	57%	[49%- 65%]	53%	[46%- 59%]	45%	[38% -52%]	65%	[57%- 71%]	1.21	[0.99–1.46]	0.81	[0.65–1.01]
(μg/l)	0.2	24%	[18%- 32%]	85%	[80%- 89%]	52%	[40%- 64%]	63%	[57%- 68%]	1.62	[1.07–2.46]	0.89	[0.80–0.98]
	0.5	8%	[5%- 14%]	99%	[97%- 100%]	87%	[59%- 98%]	62%	[57%- 67%]	9.71	[2.6–58]	0.92	[0.87–0.96]
Copeptin	14	67%	[59%- 74%]	45%	[39%- 52%]	45%	[39%- 52%]	67%	[59%- 74%]	1.22	[1.04–1.44]	0.73	[0.56–0.95]
(pmol/l)	50	33%	[26%- 40%]	79%	[73%- 83%]	51%	[40%- 61%]	64%	[58%- 69%]	1.52	[1.09–2.13]	0.86	[0.75–0.97]
	100	24%	[18%- 31%]	92%	[88%-95%]	66%	[52%- 78%]	64%	[58%- 69%]	2.91	[1.76–4.96]	0.83	[0.75–0.91]

PPV, positive predictive value; NPV, negative predictive value; LR+, positive likelihood ratio; LR-, negative LR.

A multivariable logistic regression was performed with pre-specified variables. “Epilepsy” as a variable was not included because it was correlated with the variable “first seizure” (R^2^ = 0.66). We kept “first seizure” instead of “epilepsy” in the model, because the diagnosis of epilepsy can be more subject to diagnostic disagreement than a “first seizure”. Two models are presented; one not including and the other including the biomarkers. In the first model independent risk factors for the primary outcome were found to be higher age; complex partial seizure; provoked seizure; and first seizure (Table 4). Discrimination of the model was good with a c-statistic of 0∙77 [95% CI 0∙72 to 0∙81] and Hosmer-Lemeshow goodness-of-fit test had a p = 0∙51. Bootstrap sampling confirmed the internal validity of the model, with an optimism of 0.01, and a corrected c-stat of 0.76 When adding S100-B and copeptin, the model was left unchanged, and neither of the two biomarkers was independently associated with the primary endpoint (c-stat 0.78, optimism 0.02, corrected c-stat 0.76).

**Table 4:** Adjusted odds ratios of independent predictors for composite endpoint.

**Variables**	**Adjusted ORs**	**95% CI**	**Variables**	**Adjusted ORs**	**95% CI**
Provoked seizure	4.93	2.47–9.84	Provoked seizure	4.71	2.32–9.56
Complex partial	4.09	1.84–9.08	Complex partial	4.26	1.90–9.52
First seizure	1.83	1.10–3.02	First seizure	1.73	1.03–2.89
Age (per 10 year older)	1.27	1.11–1.45	Age (per 10 year older)	1.26	1.11–1.45
			S100B	3.89	0.80–18.9
			Copeptin	1.00	1.00–1.00

OR, odds ratio; CI, confident interval.

a) clinical model, Hosmer-Lemeshow goodness-of-fit statistics p value 0.5, c-stat 0.77.

b) model with S100-B and copeptin, Hosmer Lemeshow goodness-of-fit statistics p value 0.04, c-stat 0.78.

With a modified primary endpoint that excluded those with hospitalisation for less than 24 hours, there was no improvement in terms of diagnostic performances for either of the two biomarkers. The clinical model of logistic regression showed one supplemental variable independently associated with the endpoint: pre-existing neuromuscular impairment (OR 11∙9 [95% CI 1∙44–98∙60]).

Finally, the subgroup of participants that were not admitted following their ED visit was analysed. There were 263 participants (69%) that were discharged home from the ED. Amongst them, 30 (11%) met the primary endpoint within seven days. Values of S100-B and copeptin were similar in the two groups, with a median of respectively 0∙09 μg/l and 17 pmol/l. Complex partial seizures was the only significant predictor of increased risk of recurrence (OR 5∙7 [95% CI 1∙96–16∙7]). No association was found between the level of S100-B or copeptin and the rate of secondary endpoints—only copeptin was associated with ICU admission at day seven and 28 (Table 5).

**Table 5:** Median of S100B and copeptin, with their 25%-75% interquartile range.

		**Day seven**		**Day seven**		**Day 28**		**Day 28**	
		No recurrence	Recurrence	No ICU admission	ICU admission	No recurrence	Recurrence	No ICU admission	ICU admission
S100B	(μg/l)	0.1 [0.07–0.16]	0.09 [0.06–0.17]	0.1 [0.07–0.16]	0.1 [0.08–0.20]	0.1 [0.07–0.18]	0.09 [0.06–0.15]	0.09 [0.06–0.16]	0.11 (0.08–0.20]
Copeptin	(pmol/l)	19 [8.3–54]	18 [5.2–48.5]	19 [8–53]	33 [8.2–296]	23 [9.9–66.2]	17 [0–47]	20 [8.6–54]	74 [11.1–311]

ICU: Intensive Care Unit.

## Discussion

With this study, we aimed to determine whether S100-B and copeptin are of added prognostic value to usual assessment following seizure. The first result from our study is a negative result: measurement of S100-B and copeptin has no significant added value to predict the risk of seizure recurrence or severe outcome. We found that the primary endpoint was more frequent than we expected with a rate of 40%. Finally, we present four independent clinical factors that are associated with a significant increased risk of adverse events after a seizure: higher age; acute symptomatic seizure; complex partial seizures; and a first seizure.

Although the long term rate of recurrence is well known, there is scarce data on the risk of early seizure recurrence. In its last clinical policy on evaluation of adults presenting with seizures, the American College of Emergency Physicians [31] tried to identify patient that do not need to be admitted to prevent adverse events. In contrast with literature regarding long term outcome, their level C recommendations lack studies focusing on early recurrence. As stated by Huff et al., the immediate need for admission and observation after ED evaluation has not been specifically addressed [31]. We chose a composite endpoint of early complications after ED visit that included seizure recurrence; hospital admission; death within seven days; or return visit to hospital within seven days. We consider these endpoints to be sufficiently severe that they merited being addressed collectively. The timeframe of seven days is consistent with previous literature [32,33].

In recent years S100-B has been reported to have a very high specificity for death (95% to 98%) and unfavourable neurological outcomes (85 to 98%) [34], and a very high sensitivity for the diagnosis of brain lesions (99 to 100%) [16,35] in traumatic brain injury. In the context of seizure, we report very low diagnostic performances of S100-B, with failure to obtain thresholds that would allow greater sensitivity with acceptable specificity, or vice versa. There was a very high rate of S100-B false positive (47% and 15% for a respective threshold of 0∙1 and 0∙2 μg/l), i.e. S100-B was raised in many cases that did not meet the primary endpoint. This suggests that there is a pathophysiological increase in blood concentration of S100-B after a seizure, regardless of whether that patient will go on to develop the primary endpoint or not. Similarly, we report no added value of copeptin in the setting of convulsive seizure. We failed to determine a threshold of S100-B or copeptin value that can help the clinician either to rule in or exclude the occurrence of adverse events.

The high frequency of the primary endpoint is in contrast to previously published work. This could be explained by the fact that our endpoint is a composite whereas previous studies report singular primary endpoints such as seizure recurrence. In their study in France, Choquet et al. found an early seizure recurrence rate of 19% (within 24 hours) [13], and Breen et al. suggested that a rate of at least 28% patients that were not initially admitted experienced the endpoint in the next six weeks [36]. In our study, more than a tenth of patients who were initially not admitted had an early seizure recurrence or re hospitalization within seven days.

The four independent factors we found to be associated with a significantly increased risk of adverse events after a seizure were higher age; provoked seizure; complex partial seizures; and first seizure. Besides higher age, those three conditions can contribute to the overall risk assessment a physician makes when encountering a patient that has just had a seizure. Other factors reported in the literature as carrying an increased risk of recurrence are a higher blood glucose level, a decreased GCS, and a context of alcoholism. We confirmed the influence of blood glucose level although only in the univariate analysis. However, we did not find that a decreased GCS was associated with the occurrence of adverse events—probably due to a lack of power. We also found that provoked seizure (therefore including those in the context of alcohol) is an independent risk factor of recurrence and severe outcome. This is a very valuable result as most previous studies focused on the risk of long term recurrences, and reported that provoked seizures have a lower rate of recurrence at three years (30 vs 50–70% [9–12,26]).

### Limitations

Our study has some limitations. There is a significant difference in the rate of the endpoint between France and UK. There may be inclusion bias as the ED systems of the two countries are markedly different: in the UK centre, less severe patients were managed in a different part of the ED (out-of-hours general practitioners’ clinic, or minors unit) where recruitment did not take place. Another limitation was the choice of our composite endpoint that included subjective data such as “hospitalization”. However, we determined that inclusion of hospitalization was not a serious shortcoming by running a sensitivity analysis with modified composite endpoints (with the exclusion of patients hospitalized less than 24 hours for example, and focusing only on critically ill patients), and the conclusions remained the same. Finally, there may be an element of inclusion bias because the diagnosis of seizure may be uncertain in the ED, and consequently we may have included some patients that did not have a true epileptic seizure, and may have had a pseudo-epileptic seizure or convulsive syncope. This limitation is inherent to the design and reflects the day to day work of an emergency physician, in which it is sometimes impossible to fully confirm than an epileptic seizure has occurred. In the same way, the collected data on the type of seizure were made upon patient and witness interrogation, and are consequently subject to bias. This again mirrors the real life information to which a clinician has access. A third of patients had no witness account of their seizure. To avoid inconsistencies in classification of seizure type, we classified any seizure with loss of consciousness as generalised although some of them could have been absence or focal seizure with lost of consciousness.

### Conclusion

In summary, S100-B and copeptin have very low added value to predict adverse events after an ED visit for seizure. We report four independent clinical predictors of early seizure recurrence and severe outcome: higher age; provoked seizure; complex partial seizure; and first seizure. Since the rate of adverse events is high (40%) we suggest that these conditions should alert emergency physicians to increased risk and lower the threshold for admission to the hospital.
